# A study on the implementation fidelity of the performance-based financing policy in Burkina Faso after 12 months

**DOI:** 10.1186/s13690-017-0250-4

**Published:** 2018-01-11

**Authors:** Oriane Bodson, Ahmed Barro, Anne-Marie Turcotte-Tremblay, Nestor Zanté, Paul-André Somé, Valéry Ridde

**Affiliations:** 1 ARC Effi-Santé, Political Economy and Health Economy, Faculty of Social Sciences, University of Liege, Place des orateurs, 3 (B31) – Quartier Agora, 4000 Liege, Belgium; 2 Action-Gouvernance-Intégration-Renforcement Association/Groupe de travail en Santé et Développement (AGIR/SD), Ouagadougou, Burkina Faso; 3 University of Montreal Public Health Research Institute (IRSPUM) and University of Montreal School of Public Health (ESPUM), 7101 avenue du Parc, 3rd Floor, Montréal, Québec, H3N 1X9 Canada; 4 CEPED, IRD, Université Paris Descartes, INSERM, équipe SAGESUD, 45 Rue des Saints-Pères, 75006 Paris, France

**Keywords:** Performance-based financing, Developing countries, Health financing, Implementation, Fidelity, Burkina Faso

## Abstract

**Background:**

Performance-based financing (PBF) in the health sector has recently gained momentum in low- and middle-income countries (LMICs) as one of the ways forward for achieving Universal Health Coverage. The major principle underlying PBF is that health centers are remunerated based on the quantity and quality of services they provide. PBF has been operating in Burkina Faso since 2011, and as a pilot project since 2014 in 15 health districts randomly assigned into four different models, before an eventual scale-up. Despite the need for expeditious documentation of the impact of PBF, caution is advised to avoid adopting hasty conclusions. Above all, it is crucial to understand why and how an impact is produced or not. Our implementation fidelity study approached this inquiry by comparing, after 12 months of operation, the activities implemented against what was planned initially and will make it possible later to establish links with the policy’s impacts.

**Methods:**

Our study compared, in 21 health centers from three health districts, the implementation of activities that were core to the process in terms of content, coverage, and temporality. Data were collected through document analysis, as well as from individual interviews and focus groups with key informants.

**Results:**

In the first year of implementation, solid foundations were put in place for the intervention. Even so, implementation deficiencies and delays were observed with respect to certain performance auditing procedures, as well as in payments of PBF subsidies, which compromised the incentive-based rationale to some extent.

**Conclusion:**

Over next months, efforts should be made to adjust the intervention more closely to context and to the original planning.

**Electronic supplementary material:**

The online version of this article (10.1186/s13690-017-0250-4) contains supplementary material, which is available to authorized users.

## Background

Results-based approaches (RBAs) are expanding fast in low- and middle-income countries. In particular, performance-based financing (PBF) is advanced by some as a solution to contribute to improving health system performance and thereby, achieving Universal Health Coverage (UHC) [1]. Despite its proliferation on a global scale, the research on PBF has thus far been lacking [2], only flirting with observed effects. Indeed, the great majority of studies have focused on demonstrating PBF results [3–6], without explaining the presence of both positive and negative effects. More research is still needed to understand the ‘how and why’ [7] of these effects, as shown by Ssengooba et al. [8]. In fact, very few studies have systematically investigated the implementation of PBF [2, 8–13].

Implementation evaluation is however important, if not essential. It is used to identify which elements were implemented as planned and which were not, discern the intervention’s strengths and weaknesses, and study its internal validity by assessing the cause and effect relationship. In addition to contributing actively to continuous improvement of the intervention [14], this facilitates the interpretation of an intervention’s implementation and its results. Hence, it strengthens its internal validity [15, 16] and helps avoid Type III errors in evaluation that would lead to mistakenly attributing a lack of effects to the intervention itself without considering the quality of its implementation. The premise underlying the study of implementation fidelity is that a program’s implementation influences its effectiveness. The appropriate degree of fidelity, however, is the subject of debate between defenders of total fidelity to the theoretical model and proponents of essential adjustments. Deviating from the plan is seen by some as compromising the program’s effectiveness [17] and by others as key to making necessary adjustments to contextual factors surrounding the intervention [18, 19]. Nevertheless, all agree there are certain core elements specific to each program that are essential to ensure its effectiveness implemented and that these must be implemented with fidelity [18, 20].

Thus, the aim of the present study was to deepen our understanding of the “black box” of PBF, taking Burkina Faso as a case study. In Burkina Faso, the achievement of UHC continues to be compromised by insufficient health funding and poor health system performance [21], with child and maternal mortality rates among the highest globally [22]. The situation prompted the Ministry of Health (MOH) to undertake a PBF intervention through a World Bank(WB)-supported project, as a trial project in three districts first in 2011 and since 2014 under the form of a three-year pilot project. Our objective was to analyze the implementation fidelity of the PBF pilot project in Burkina Faso in order to understand and assess the degree of implementation 12 months post-launch.

PBF was introduced in Burkina Faso as a trial project in the districts of Boulsa, Léo, and Titao over nine months, from April to December 2011. Its results, deemed “encouraging” by evaluators [23], justified its expansion on a larger scale. In 2014, the PBF mechanism took the form of a three-year pilot project funded by the WB. That intervention affected all echelons of the health system, which is organized as a three-tiered pyramid providing primary, secondary, and tertiary care. The base tier consists of 1698 health and social promotion centers (CSPS - *centre de santé et de promotion sociale*) and 47 medical centers with surgical units (CMA - *centre médical avec antenne chirurgicale*). The second tier consists of 9 regional hospitals (CHR – *centre hospitalier régional*), and the third, 4 university hospitals (CHU - *centre hospitalier universitaire*) [24]. Altogether, the PBF intervention covered four of the fourteen health regions encompassing 15 districts, 11 CMAs and 561 CSPSs. In some health districts (HD) its implementation was coupled with a community-based indigent-selection intervention and a community-based health insurance (CBHI) program, to influence the service demand side as well.

At the CSPS level, the present PBF exercise was designed as a randomized controlled trial. The four randomization categories were: 1) PBF 1: the health center is paid for indicators of activities performed, based on contractually set prices; 2) PBF 2: same as PBF 1, coupled with an intervention for community-based selection of indigents to be exempted from user fees; 3) PBF 3: same as PBF 2, with an additional subsidy (“equity bonus for indigent care”) to encourage initiatives aimed at increasing service use by indigents; and 4) PBF 4: same as PBF 1, to which is added a community-based insurance plan coupled with community-based indigent selection. This last component of PBF was implemented only in the Boucle de Mouhoun health region.

The principle underlying PBF is that health centers are remunerated based on the quantity and quality of services they provide based on a matrix of quantitative indicators along with other quality measures, the whole formalized in a contract between the health center and a designated contracting and auditing agency. In the context of Burkina Faso, the provision of services is monitored by means of monthly audits of quantitative indicators, quarterly audits of quality measures, community-based (local) audits of the accuracy of information entered into health center registers, and user satisfaction surveys. The quantitative and qualitative audits are subsequently cross-audited in a sample of health centers. PBF financial incentives are distributed based on the results of those audits. The funding received is intended to cover health center expenses, and a maximum of 30% may also be paid to staff in the form of individual performance bonuses. Thus, the process is based on the key premise that financial incentives are effective in improving the quality and quantity of health services [25]. Health centers may also qualify for three additional bonuses: 1) the “quality improvement” bonus, to cover expenses associated with improvements to infrastructure and equipment; 2) the “inter-district and inter-health center equity” bonus, which is intended to compensate for inequalities by adjusting the price associated with the quantitative indicator based on a classification established between health districts and between health centers within a same district^1^; and 3) the “indigent care” bonus, specific to PBF 3, which financially compensates health centers that provide care to indigents who, upon presentation of their card, do not pay for medical care or medications. In practice, this compensation takes the form of a higher purchase price for certain indicators as ambulatory consultations for patients aged five years and over with physicians, specialized nurses, or state-certified midwives (female or male) and patient admissions to hospital.

## Methods

### Analysis framework and activities analyzed

This study was part of a larger research project designed as a multiple case study with several embedded levels of analysis [26]. The selection of cases is presented and explained elsewhere [27]; for the present study we retained, in three health districts, 1 CHR, 2 CMAs, and 18 CSPSs.

Our study was based on the implementation fidelity analysis framework of Carroll et al. [28], which is considered to be particularly comprehensive [27]. We first compiled an exhaustive list of all activities planned based on the intervention’s official documentation (as implementation guide, action plans and meeting reports). For each activity listed, we specified the content, coverage, and temporality to assess whether the “active ingredients” of the intervention have been received by the “beneficiaries” where, as often and for as long as it should have been [28]. The term *content* refers to the activity that was implemented and is under analysis. *Coverage* refers to the public affected by the activity. *Temporality* refers to the timing, or time frame, of the activity implemented.

We therefore began with an exhaustive compilation of this list based on four dimensions identified through a full review of all activities: 1) planning (training workshops and recruitment); 2) application (intervention launch, performance audits, determination and payment of subsidies); 3) tools (purchase contracts, reporting systems); and 4) action research. We then made a careful selection of activities considered to be core, understood as fundamental to the intervention’s effectiveness [18, 20]. Thus, for instance, activities having to do with supplying office materials and furniture for technical services supporting the PBF implementation at the central level were not retained. Another such example is the analysis of data related to the introduction and payment of bonuses for indigent care, which referred only to the 5 CSPSs classified as PBF 3 (*n* = 5), as they were the only ones receiving these bonuses. Thereafter, we grouped certain core activities together to facilitate understanding and use of the list. This list of core activities was finally submitted to and validated by the authorities in charge of the PBF program who recognized the listed activities as essential.

### Data collection

Data were collected at two points in time to identify: 1) the activities planned and 2) the activities that had been implemented after one year of operation. To construct the list of planned activities, we analyzed in-depth all official documents available as of March 31, 2014. Then, in February and March 2014, we interviewed eight key informants who knew the intervention well, using a non-random sampling approach for which the inclusion criteria (*criterion-i*) were position held, availability, and knowledge of the intervention [29].

The empirical data were then collected 12 months after the intervention’s launch, between December 2014 and February 2015. One year was considered to be sufficient time for the stakeholders to have installed the intervention. The data were obtained from interviews with key actors involved in implementing PBF in each of the 21 health centers (CHR, CMAs, CSPSs). Two sampling strategies were applied based on the type of care facility. First, the CSPS sample included directors of centers, managers of drug depots, health workers (assistant head nurses, maternal care managers, managers of nursing curative consultations, managers of expanded immunization programs), and available support personnel. Then, given the limited availability of CMA and CHR personnel, the preferred approach was to speak with the head of the facility and anyone else available who knew about the PBF program implementation.

Two interview techniques were used, depending on the number of resource persons available. Focus groups were conducted in the primary care facilities (CSPSs and CMAs) and individual interviews in the secondary care facility (CHR). In all, 83 resource persons were interviewed: 76 persons in 18 focus groups in the CSPSs, six persons in two focus groups in the CMAs, and one individual interviewed in the CHR.

Persons interviewed were invited to react to all the activities listed for their level and tier of care. For each activity, respondents were asked to provide a substantiated and detailed assessment of its implementation in terms of three statuses: implemented as planned, not implemented as planned, or modified. They could also mention other activities that had been added. The ethnographic notes and logbooks of the survey team were also used as sources of empirical data and help a very few times to correct the status picked when it was not coherent with the health worker’s statement. All the data were entered into a matrix encompassing the different dimensions studied.

### Data analysis

The data were analyzed following the analysis framework method [30] using the framework adapted from Caroll et al. [28]. Qualitative data regarding activity content fidelity were translated into quantitative data by considering the proportions of activities categorized by respondents as: 1) implemented as planned; 2) not implemented as planned; 3) modified; and 4) added. A fifth option, “non-response” (NR) was added to take into account missing responses. Our approach can be illustrated by taking as an example the activity of receiving tools for reporting purposes. Of the 21 health centers studied, 19 reported they had implemented the activity according to plan, one saw the activity modified, and one reported it was not implemented. The activity was therefore predominantly implemented as planned (90%, 19/21) and presented low rates of modification (5%, 1/21) and of non-implementation (5%, 1/21). Temporal fidelity was studied in terms of frequency (e.g. monthly, quarterly) and, when the activity allowed, duration, understood as number of days planned for the activity.

## Results

### Content

Regarding the content of the intervention implemented, we observed that the majority of the intervention components were implemented with fidelity (65.5%, 249/380), while 25.8% (98/380) were not implemented and 7.4% (28/380) underwent modifications (NR: 1.3%, 5/380). Thirteen activities (3.4%, 13/380) were added during the implementation because of contextual circumstances. For example, a training activity on the PBF portal was added at the CHR, which had not been planned. More than half of the added activities (61.5%, 8/13) occurred during the planning process and consisted of information sessions and recruitment activities. They were fully implemented (8/8). The remaining 38.5% (5/13) involved the application dimension, and in particular, the performance audit process. These latter activities presented a lower rate of implementation (60%, 3/5), with only two of the five activities added to the application dimension having ultimately not been implemented.

The highest implementation rate was seen in the planning dimension (91.2%, 31/34) (Table 1), which encompassed training workshops and recruitment activities. Training activities were, as a whole, implemented with fidelity. The next highest implementation rate was reached by reporting and auditing tools reception activities (tools dimension) (69.8%, 44/63). Tools for reporting and for quality auditing appeared to have been received with relatively few problems, as this the activities related showed implementation fidelity of 88.1% (37/42). Furthermore, health centers encountered certain difficulties in reinforcing their performance improvement plans. Just one-third (33.3%, 7/21) of the activities to improve these plans had been implemented with fidelity. The application dimension was implemented with fidelity in 65.3% (171/262) of cases, even though it represented in some ways the flagship activities of the PBF intervention, i.e., performance auditing and the determining and payment of subsidies. The action research dimension was especially under-implemented (14.3%, 3/21). In fact, few such actions were initiated, primarily due to poor communication on the nature of the activity, but also because the activity was to be undertaken at each health center’s convenience.

**Table 1:** Content fidelity of the implementation by dimension

	Planning(*N* = 34)	Application(*N* = 262)	Tools(*N* = 63)	Action research(*N* = 21)
Implemented as planned	91.2%	65.3%	69.8%	14.3%
Not implemented	0.0%	26.0%	20.6%	81.0%
Modified	8.8%	7.3%	7.9%	4.8%
No response	0.0%	1.5%	1.6%	0.0%

**Fig. 1:**
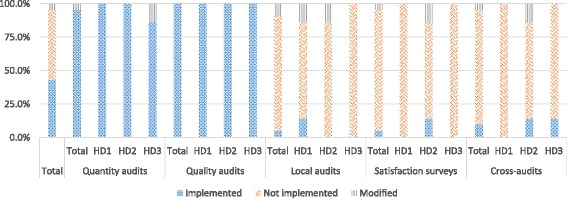
Comparison of the degrees of implementation of performance audits among health districts (HD)

Within the application dimension, performance auditing activities were, generally speaking, poorly implemented (42.9%, 45/105). Closer examination showed clear heterogeneity (Fig. 1). While quantitative and qualitative audits were very widely implemented, at 95.2% (20/21) and 100% (21/21) respectively, that was not the case for local audits, audits based on satisfaction surveys, and cross-audits, all of which encountered significant implementation failures. The implementation rate for local audits and satisfaction surveys was 4.8% (1/21), and for cross-audits, 9.5% (2/21). The low implementation rate for local audits of the accuracy of information recorded in the registers was primarily due to delays in recruiting surveyors, as the body responsible for organizing this recruitment had not yet been trained across all sites. This lack of surveyor recruitment also had repercussions on satisfaction surveys, which were conducted in tandem with local audits. Furthermore, in instances where local audits were carried out, the target sample of 40 to 60 persons randomly drawn from the health area was not always attained, nor was the random nature of the sampling always respected. For example, in one health center the local audit used a sample of only 20 mothers. Other audits were also affected. The delays caused by inadequate recruitment gave rise to several types of compensatory strategies, such as using temporary medical auditors for quantitative auditing (before the designated contracting and auditing agency was put in place), and using this same auditing agency to replace the external structure recruited to conduct cross-audits.

**Fig. 2:**
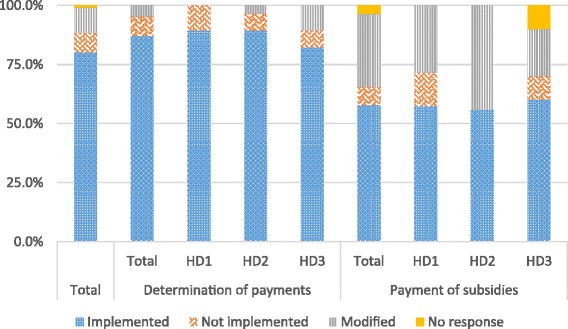
Comparison of implementation fidelity of activities related to determination and payment of subsidies by HD

Unlike performance auditing activities, activities related to the determination and payment of PBF subsidies showed higher levels of implementation fidelity (80.0%, 88/110) (Fig. 2). Still, implementation fidelity rates differed between activities involved in determining subsidies—i.e., production and transmission of results of quantitative and qualitative audits—and activities involved in paying those subsidies, at 86.9% (73/84) and 57.7% (15/21), respectively. With regard to the specific bonuses, two health centers received a “quality improvement bonus” and two received an “inter-district and inter-health centre equity bonus”. The bonus for providing care to indigents that was intended for PBF 3 centers was not distributed as planned (0%, 0/5).

### Coverage

We assessed to which extent core activities are implemented across health districts and observed that the three health districts (Table 2) present very similar coverage statistics, even though there were more modifications to activities in the second district (11.1%).

**Table 2:** Fidelity of implementation content by health district (HD)

	HD1(*N* = 124)	HD2(*N* = 126)	HD3(*N* = 130)	Total(*N* = 380)
Implemented as planned	68.5%	64.3%	63.8%	65.5%
Not implemented	28.2%	23.8%	25.4%	25.8%
Modified	3.2%	11.1%	7.7%	7.4%
No response	0.0%	0.8%	3.1%	1.3%

**Fig. 3:**
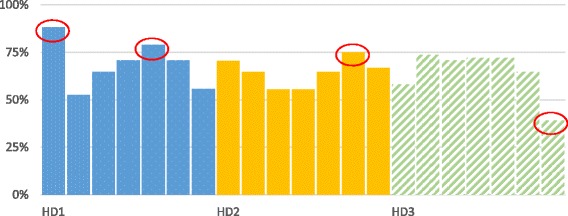
Comparison of implementation by HD

Figure 3 presents the degree of implementation for each health center in our survey in the three districts and reveals in this way the degree of coverage across health facilities. The figure shows a certain internal heterogeneity. Several health centers presented extreme implementation percentages (circled in red): under 50% for one and more than 75% for three others.

**Fig. 4:**
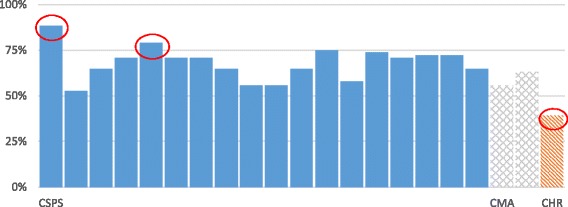
Comparison of implementation by tier and level of care

Further, implementation fidelity was greater in the CSPSs (68.2%, 219/321) than in the CMAs (58.3%, 21/36) or the CHR (44.0%, 11/25) (Table 3). There were, however, significant disparities among the CSPSs, shown in blue (Fig. 4). Also, a high percentage of modifications was noted at the CHR level (30.4%, 7/23).

**Table 3:** Fidelity of implementation content by tier and level of care

	CSPS(*N* = 321)	CMA(*N* = 36)	CHR(*N* = 23)
Implemented as planned	68.2%	58.3%	39.1%
Not implemented	25.5%	33.3%	17.4%
Modified	5.9%	5.6%	30.4%
No response	0.3%	2.8%	13.0%

**Fig. 5:**
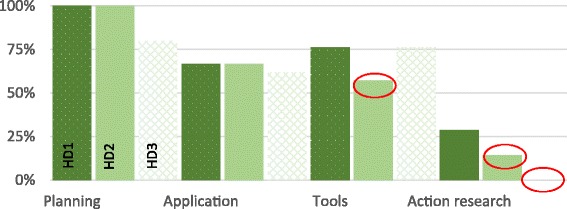
Comparison of implementations by dimension and by HD

Such heterogeneity was equally present in the study of content fidelity by dimension and by district (Fig. 5). The second district presented particularly low implementation statistics for the tools reception activities compared to the other two districts. As for the action research dimension, which was scarcely implemented, strong disparities were observed among districts, with little and no implementation in the second and third districts, respectively.

There was relatively strong homogeneity among health districts in the implementation of performance audit activities. The second district presented, overall, the greatest number of modifications with respect to performance audit activities (Fig. 1).

For activities related to the determination and payment of subsidies, implementation fidelity was evenly distributed among the three districts (Fig. 2). Modification rates varied by health district.

### Temporality

Assessing the temporality of an activity allows determining the time frame under which the activity was carried out and comparing it to the original plan. Overall, 63.4% (241/380, NR: 1.1%) of activities adhered to the planned schedule, but 14.2% (40/280) of the activities implemented were altered. Temporal disparities were most often observed in HD2 (17.0%, 16/94), as opposed to 12.0% (11/92) in HD1 and 13.7% (13/95) in HD3, and in the application dimension, where 18.5% (30/162) of the activities implemented were altered. Given the low implementation fidelity for this dimension, at this stage we can only present a few nuances. The activities most affected by temporal disparities were performance audit activities (42.9%, 45/105) and payment of subsidies (57.7%, 15/21). Exceptions were the quantitative (95.3%, 20/21) and qualitative (100%, 21/21) audits, which were largely conducted according to schedule. One health center reported, however, that it was subjected to a quarterly audit after only one month of implementation, due to delays in launching the intervention. Conversely, the activities most often altered—if they were even conducted—were local audits (0%, 0/3), satisfaction surveys (33%, 1/3), and cross-audits (50%, 2/4). Most often, these numbers were due to implementation delays. Still, one health center advanced its local audit by one quarter (T2 2014 instead of T3 2014). With respect to subsidies, the duration of activities to calculate payments conducted at the facility level was often brief, at one day.

## Discussion

The empirical data showed most planned activities were implemented in accordance with the national plan. However, more detailed examination offers a more contrasted view, which is very reasonable in such a context, with implementation discrepancies being inevitable [31] and already observed elsewhere [11, 12, 32]. Disparities between the plan and the implementation varied considerably according to the components under study. The planning dimension, which encompassed both recruitment and training activities, was generally implemented with fidelity. The same was true for reception of the performance implementation tools, which appeared to have transpired with no major difficulty. Most bottlenecks were related to performance auditing and payment of subsidies, as previously observed in Sierra Leone [33, 34], Nigeria [11] and in Benin [12].

This situation is also very familiar to African countries, which adopt user fees exemption policies and then experience significant delays in health center reimbursements [35–38]. In fact, in the case of PBF in Burkina Faso, local audits, audits based on satisfaction surveys, and cross-audits all encountered significant implementation gaps. The same was also true for incentive bonuses, which were infrequently paid even though they had been mainly calculated in the majority of cases.

The observed gaps in these two components after one year of implementation are worrisome because these mechanisms are at the heart of PBF intervention theory. The risk is twofold. First, delays in incentive bonuses threaten the intervention’s credibility by undermining the trust of care providers [39] and creating, as in Sierra Leone [33, 34], Nigeria [10, 11, 40], Congo [41], and even India [42], frustrations that have a deterrent effect [43], thwarting the objective of motivating care providers. In Nigeria, for example, payment delays created a climate of uncertainty among health workers regarding the promised subsidies [10, 11]. As doubts took hold, care providers preferred to focus on activities with immediate benefits to themselves rather than make additional efforts for illusory subsidies [10]. Moreover, such delays can undermine the intervention’s ability to achieve its intended purpose of improving health services quality and quantity. In Uganda, Ssengooba et al. [8] showed, in fact, that excessive delays led to loss of institutional memory regarding the PBF intervention, and particularly regarding performance targets, thereby impeding achievement of the intended outcomes.

This situation is not unique to PBF. Olivier de Sardan and Ridde [37], in studying user fees exemption policies, also found that payment delays jeopardized the outcomes by giving rise to diversionary tactics focused on more profitable procedures.

Furthermore, the fact that no health center in our study implemented the PBF intervention entirely according to plan adds fuel to the current debate about the adaptability of interventions, including PBF. On one side are the proponents of absolute adherence to the theoretical model and, on the other, advocates of a relative compliance in which core elements are retained [18, 20]. The latter maintain that such adaptations are needed so that context is taken into account, to ensure the intervention’s effectiveness and viability [18, 19, 44]. In Uganda, Ssengooba et al. [8] observed, in fact, that a lack of financial resources prompted certain adaptations in order to achieve the intended outcomes despite budget cuts. In Rwanda, the performance assessment grid was modified twice, in 2008 and 2010, to adapt the PBF intervention more closely to the changing needs of the hospital sector, that is, to bring the grid in line with stricter quality norms [45].

In our study, 7.4% (28/380) of activities had been modified and 3.4% (13/380) had been added, for a combined intervention adaptation rate of 10.8%. However, it would seem that is not so much the adaptation rate that should be examined, as the nature of these adjustments. Rebchook, Kegeles, Huebner, and the TRIP Research Team [46], cited by Perez et al. [47], identified three typologies of adaptations based on their secondary effects: 1) adaptations that profoundly alter the intervention to the point where it no longer produces the intended outcomes; 2) minor or major adaptations that do not impede fidelity and may even make the intervention more effective; and 3) added activities that do not affect implementation fidelity a priori. More concisely, some adaptations are deemed “acceptable” and others “risky” or even “unacceptable” [48], and some are considered to have positive or negative effects, or none at all, on the intervention [49]. However, almost no studies have examined such adaptations. Breitenstein, Robbins, and Cowell [50] suggest these adaptations should be identified and evaluated to determine the nature of their effects. It would also be interesting, in future qualitative studies, to explore the 13 adaptations observed in our study and their effects on the intervention.

The main strength of our study lies in the originality of its subject. In fact, the current literature on PBF is focused primarily on impact evaluations to demonstrate effects (positive and negative), but without attempting to understand them. In our case, the results can be used to support subsequent analysis of the implementation process and intervention outcomes. Our study does, however, present certain limitations. The first has to do with a weakness in the sample. Only two CMAs and one CHR were considered in our study, thereby precluding any extrapolations; they did, however, provide a view of the situation that remains to be confirmed or countered in our next studies. This is especially important given the scarcity of studies on PBF in hospitals in Africa. A second limitation emerged during data collection, with respect to the availability of resource persons with information on the PBF intervention. In some cases, it was complicated to locate anyone who knew about it. Sometimes there was indeed only one person with knowledge about PBF, such that we conducted just one interview in that facility, which in itself provided useful information on the quality of implementation. High turnover of health personnel, poor transmission of information, and lack of institutional memory are phenomena that have been frequently observed in Africa [51]. Even when resource persons were available, they sometimes had difficulty recalling when exactly specific activities were implemented. When necessary, temporal fidelity was questioned quarterly, which helped informants to recall better. A third limitation concerns the understanding of implementation fidelity in its broadest sense. Our research objective was to determine the extent to which the PBF intervention had deviated from its planned model. This objective refers to the notion of content fidelity. However, Dumas, Lynch, Laughlin, Phillips Smith, and Prinz [52] assert that content fidelity is not sufficient to comprehend implementation fidelity as a whole, in all its subtleties. Those authors argue that process fidelity must also be considered as investigated by Ridde et al. [53], observing how the plan is implemented by those on the ground, which corresponds to “implementation quality” [19]. This angle of research merits further development for a more comprehensive picture of implementation fidelity.

## Conclusion

Twelve months post-launch, or one-third of the way through the pilot project, the implementation fidelity of the planning dimension—encompassing information, training, and recruitment activities—had provided a solid foundation for the intervention. However, delays and sometimes serious implementation deficiencies were also observed, particularly with regard to performance audit mechanisms and PBF subsidy payments. Supplementary bonuses, such as the bonus for indigent care, were also rarely distributed, or not at all. Poor implementation of these core activities of the PBF intervention is a harbinger of delays in intended outcomes and could, more broadly, even jeopardize its viability. The intervention is, however, still in its early days. There are many months left in which the intervention can be more closely adapted to both the context and the original plan. More fundamentally, we take the opportunity provided by this study to point out that the field of implementation fidelity research has thus far produced very little literature, and we invite global health researchers to consider the significance and advantages offered by such an angle of study as it pertains to intervention effectiveness [54]. Clearly, such fidelity analysis is necessary, but not sufficient. More research needs to be developed and conducted to better understand the PBF implementation process in Africa, as well as its impacts (intended or not) and their heterogeneity.

## Additional file

Additional file 1: Dataset. (XLSX 19 kb)

## Footnotes

1 Bonuses create variations in the price of the quantitative indicator ranging from +0% to +40%, depending on the district, with a further +0% to +40%, depending on the health center. Thus, the base price for an assisted delivery is set at 1.500 F CFA, but a health center classified at +20% (inter-health center equity bonus) and located in a district classified at +30% (inter-district equity bonus) would receive 2.250 F CFA for the procedure.
